# Sex Differences in the Association between Prolonged Sitting Time and Anxiety Prevalence among Korean Adults

**DOI:** 10.3390/brainsci14070729

**Published:** 2024-07-20

**Authors:** Eunsoo Kim, Chul-Hyun Park, Hyun-Seung Lee, Mi Yeon Lee, Sung Joon Cho

**Affiliations:** 1 Department of Psychiatry, Kangbuk Samsung Hospital, Sungkyunkwan University School of Medicine, Seoul 03181, Republic of Korea; silverstream421@gmail.com; 2 Department of Physical and Rehabilitation Medicine, Kangbuk Samsung Hospital, Sungkyunkwan University School of Medicine, Seoul 03181, Republic of Korea; chpark0930@gmail.com (C.-H.P.); hyunseung11.lee@samsung.com (H.-S.L.); 3 Department of Biostatistics, Kangbuk Samsung Hospital, Sungkyunkwan University School of Medicine, Seoul 03181, Republic of Korea; my7713.lee@samsung.com; 4 Workplace Mental Health Institute, Kangbuk Samsung Hospital, Seoul 03181, Republic of Korea

**Keywords:** prolonged sitting time, anxiety, sex, mental health

## Abstract

Sex differences in the effect of prolonged sitting time on anxiety symptoms have not yet been explored. This study examined the sex-specific association between prolonged sitting time and anxiety prevalence in Korean adults. Community-dwelling adults aged >18 years who underwent a cross-sectional structured study survey of physical activity and mental health tests were enrolled as part of the Kangbuk Samsung Hospital Cohort Study from 2012 to 2019. The prevalence of anxiety was evaluated using the Clinically Useful Anxiety Outcome Scale (CUXOS) questionnaire. The mean daily sitting time was 7.9 ± 3.4 h in men and 6.8 ± 3.6 h in women. After adjustments for possible confounding factors, the adjusted mean CUXOS score was the highest in participants sitting for ≥10 h, followed by 5–9 h, and <5 h, in that order. In the post-hoc Bonferroni analysis, there were significant differences in the adjusted mean CUXOS scores in group comparisons. A multivariate logistic regression analysis was conducted after adjusting for potential confounding factors. A prolonged sitting time was positively associated with an increased prevalence of anxiety in both men and women, with stronger associations in women than in men. It is necessary to prevent anxiety by adjusting or reducing sitting time in adults, especially women.

## 1. Introduction

In modern society, prolonged sitting has become a common phenomenon in the digital era with the widespread use of computers, the Internet, mobile devices, and social media. Behind the enticing convenience of cutting-edge technology lies the risk of increased all-cause mortality due to cardiovascular diseases, diabetes, cancer, and other health conditions associated with prolonged sitting [1,2,3]. Moreover, prolonged sitting negatively affects mental health, contributing to conditions such as depression [4,5], anxiety [6], and cognitive impairment [7]. During recent COVID-19 lockdowns, when extreme social isolation and home confinement occurred, increased sitting time was also strongly linked to chronic diseases and adverse mental health outcomes [8,9]. Recognizing the independent effects of sitting tendencies and physical behavior on health indicators [1,10], the World Health Organization (WHO), in 2020, recommended limiting sedentary behavior across all age groups, irrespective of physical activity [11].

South Korea, recognized as an information technology powerhouse, led the forefront of the information and communications technology development index globally in the last decade [3,12]. With a household Internet access rate of 99.96%, digital device usage has become ubiquitous among the Korean population [13]. Additionally, South Korea is characterized by exceptionally long annual working hours, consistently ranking among the top OECD countries with approximately 2000 h over the past decade [14]. The advent of the digital era has further exacerbated the average sedentary time owing to the shift to telecommuting and the widespread use of smartphones. These South Korean characteristics are significant for illustrating the consequences of prolonged sitting times.

Globally, anxiety disorders affect a substantial proportion of the population, with a lifetime prevalence ranging from 14.5% to 33.7% in the United States [15,16]. Anxiety disorder is the second leading cause of disability-adjusted life years related to mental health [17], significantly contributing to healthcare costs and the burden of illness [18,19]. Since 2003, the burden of anxiety disorder has been on the rise to date [20]. Few studies have hypothesized that prolonged sitting may lead to anxiety through biological and psychosocial pathways [7,19,21,22,23,24,25]. For example, increased screen time with prolonged sitting affects CNS arousal and sleep cycles [7,21]. Additionally, poor metabolic health, such as obesity, can result in changes in body image, decreased self-esteem, and social withdrawal, thereby exacerbating anxiety symptoms [22]. Moreover, anxiety disorders largely coexist with chronic illness patients, such as metabolic disorders, cardiovascular diseases, diabetes, and stroke [19,23,24]. Patients with chronic illness often have increased health concerns, mobility issues, and diminished social interaction, leading to an increase in sedentary behavior [23,25]. Chronic physical condition was also linked to antidepressants and anxiolytic, hypnotic, or sedative medication use [23].

Nevertheless, the evidence regarding the relationship between prolonged sitting time and anxiety remains limited. While global research on the association between prolonged sitting time and anxiety has often combined sitting time and screen time, making it challenging to isolate the emotional effects of content consumption and the implications of addiction [7,26,27,28], studies in South Korea have been limited to specific populations such as adolescents [29], older adults [30], and university students [31], limiting the generalizability of the findings. Furthermore, sex differences in the impact of sitting time on anxiety have been overlooked despite significant variations in anxiety prevalence between sexes [18] and existing physiological and behavioral differences [25,32]. Building upon prior research, sedentary behaviors may have more detrimental effects on the physical health of women than that of men [1,33]. Thus, this study aimed to confirm the sex-differentiated association between prolonged sitting time and anxiety prevalence by exploring potential hypothetical mechanisms using health examination data from approximately 150,000 individuals.

## 2. Materials and Methods

**Figure 1:**
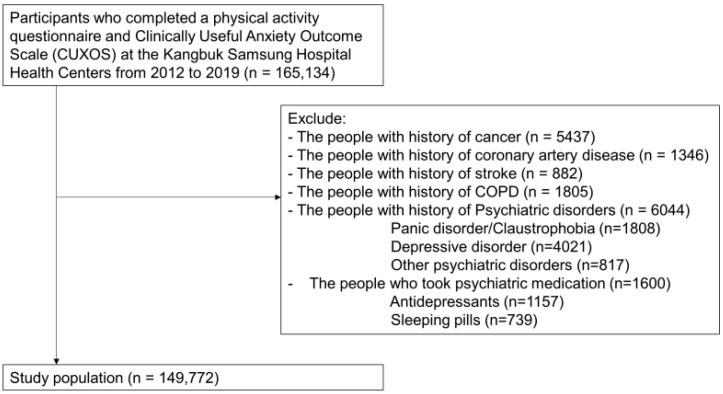
Selection of study population.

This cross-sectional study was conducted at the Kangbuk Samsung Medical Center, Seoul, and the Korea Medical Institute Suwon Center. We recruited 165,134 participants (aged >18 years) who completed the physical activity questionnaire and the Clinically Useful Anxiety Outcome Scale (CUXOS) at the Kangbuk Samsung Hospital Health Centers from 2012 to 2019. After excluding 11,046 participants who met one or more of the exclusion criteria, data from 149,772 participants (85,636 men and 64,136 women) were analyzed to determine the association between prolonged sitting time and anxiety symptoms (Figure 1). The study protocol was approved by our hospital’s Institutional Review Board (IRB; IRB No. KBSMC 2022-07-017). An IRB waiver from the requirement for informed consent was obtained because de-identified samples routinely collected as part of a health screening test were used.

### 2.1. Measurements

The Korean version of the CUXOS was used to measure the severity of participants’ anxiety symptoms. Previous studies have reported an internal consistency of 0.90 and test–retest reliability of 0.74 for the Korean version of the CUXOS. The questionnaire comprises 20 self-report items, including 6 items in the mental anxiety domain and 14 in the physical anxiety domain, and participants respond to each item using a five-point Likert scale. Each item is scored from 1 to 4, with an overall score of 0–80. A score of 20 was suggested as the optimal cut-off point when screening for clinically meaningful anxiety, consistent with previous research [34,35].

Sitting time and physical activity were measured using the validated Korean version of the International Physical Activity Questionnaire (IPAQ) Short Form [36]. The IPAQ measures the weekly duration and frequency of walking and other moderate-to-vigorous physical activities conducted for >10 continuous minutes across all behaviors (including work, home, and leisure). The total 7-day sitting time was measured using the following question: “During the last 7 days, how much time did you usually spend sitting?” To date, there are no established applicable thresholds for reference data presented as categorical levels for sitting time. In this study, sitting time was classified into the following groups using the thresholds commonly used in previous studies: <5, 5–9, and ≥10 h/day [37]. The frequency of vigorous exercise was measured using a single question: “During the last 7 days, on how many days did you do vigorous physical activities like heavy lifting, digging, aerobics, or fast bicycling?”

Information on the participants’ age, sex, screening center (Seoul or Suwon), smoking history (never, former, or current), alcohol consumption, education (high school graduate or lower, community college or university graduate), history of hypertension, diabetes mellitus, and dyslipidemia was collected using standardized questionnaires through an interview by a physician. Male and female participants with an alcohol consumption of 20 g or more and 30 g or more per day, respectively, were categorized as heavy drinkers [38].

This study measured anthropometric data including body mass index (weight (kg)/height (m)^2^), waist circumference, and body composition to adjust for the risk factor of obesity to explore the association between sitting time and anxiety [39]. Previous research has suggested that anxiety is associated with abnormal anthropometric profiles related to chronic metabolic diseases, which often coexist with sedentary behavior [40,41]. Trained nurses performed anthropometric measurements including height, weight, and body composition. The waist circumference was measured in a standing position at the level of the umbilicus. Appendicular skeletal muscle mass, fat mass, and body fat percentage were estimated using a multifrequency bioimpedance analyzer with eight-point tactile electrodes (InBody 720; Biospace Co., Seoul, Republic of Korea), which was validated regarding reproducibility and accuracy for body composition [42]. Blood samples were collected after 10 h of fasting and analyzed in the same core clinical laboratory by medical laboratory technicians. Serum biochemical parameters, such as vitamin D, total cholesterol, low-density lipoprotein cholesterol (LDL-C), high-density lipoprotein cholesterol (HDL-C), triglycerides, fasting glucose, creatinine, albumin, and aspartate aminotransferase (AST), were measured using an auto-analyzer (747 Automatic Analyzer, Hitachi, Tokyo, Japan). Hemoglobin A1c (HbA1c) was measured using an immunoturbidimetric assay with a reference value of 4.4–6.4%, aligned with the Diabetes Control and Complications Trial and National Glycohemoglobin Standardization Program standards [43].

### 2.2. Statistical Analysis

The baseline characteristics of the study population were analyzed using the chi-square test for categorical variables and the Student’s *t*-test for continuous variables. Multiple logistic regression analysis was performed to determine the association between sitting time and anxiety in both men and women. We used four models to adjust for confounding factors. Model 1 is a crude analysis without any adjustments. Model 2 was adjusted for age, screening center, alcohol intake, smoking status, and educational level. Model 3 was additionally adjusted for a history of hypertension, history of diabetes mellitus, LDL-C, waist circumference, and vitamin D. Finally, Model 4 was additionally adjusted for the frequency of vigorous exercise. Interactions between subgroups were analyzed using likelihood ratio tests to compare models with and without multiplicative interaction terms. The mean CUXOS scores between categories of sitting time (<5 h/day, 5–9 h/day, and ≥10 h/day) were compared using an analysis of covariance (ANCOVA) after adjusting for age, screening center, alcohol intake, smoking status, education level, history of hypertension, history of diabetes mellitus, LDL-C, waist circumference, vitamin D, and vigorous exercise frequency. Interactions by sex were analyzed using likelihood ratio tests to compare models with and without multiplicative interaction terms. Statistical significance was set at a two-tailed significance level of *p* < 0.05. All the analyses were performed using STATA version 17.0 (IBM Corp., Armonk, NY, USA).

## 3. Results

### 3.1. Baseline Characteristics of Study Participants

The baseline characteristics of men and women are summarized in Table 1. Among the 149,772 participants (85,636 men; 64,136 women), the means (standard deviation; SD) for sitting time were 7.9 (3.4) h/day for men and 6.8 (3.6) h/day for women. The frequency of vigorous exercise was higher in men (1.1 [1.5] days/week) than in women (0.7 [1.4] days/week). In men, the prevalence rates of comorbid conditions, such as hypertension, diabetes mellitus, and dyslipidemia (17.7%, 4.1%, and 22.1%, respectively), were higher than those in women (5.8%, 1.5%, and 9.1%, respectively). All variables differed significantly between women and men (*p* < 0.001).

### 3.2. Comparison of Adjusted Mean CUXOS Scores between Categories of Sitting Time

**Figure 2:**
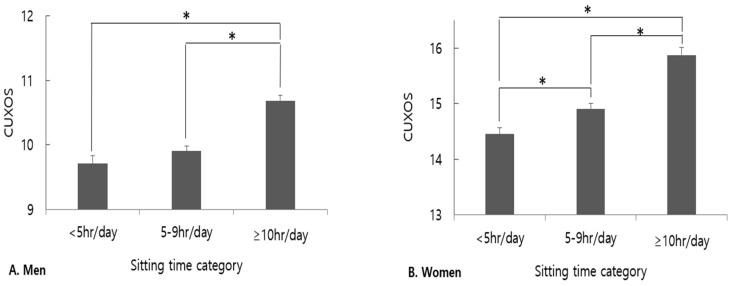
Comparison of adjusted mean of CUXOS scores between categories of sitting time (<5 h/day, 5–9 h/day, ≥10 h/day). Adjusted means (±SE) of CUXOS scores in the groups were estimated from ANCOVA after adjusting for confounding variables. * Group difference by Bonferroni post-hoc *p* < 0.05; vit D, vitamin D; LDL-C, low-density lipoprotein cholesterol; CUXOS, Clinically Useful Anxiety Outcome Scale; SE, standard error.

Figure 2 illustrates a comparison of CUXOS scores (as a continuous variable) according to sitting time for male and female participants. In the Bonferroni post-hoc analysis for group comparisons, the adjusted mean of the CUXOS scores for the sitting time of ≥10 h/day was significantly higher than those of the sitting time of <5 h/day and 5–9 h/day in male participants, (all post-hoc; *p* < 0.05) after adjusting for possible confounding factors. In women, there were significant differences in the adjusted mean of the CUXOS scores between the sitting time of <5 h/day, 5–9 h/day, and ≥10 h/day (all post-hoc; *p* < 0.05), after adjusting for possible confounding factors.

### 3.3. Sex-Differentiated Association between Sitting Time and Anxiety Prevalence

The results of the multivariate logistic regression analyses to assess the association between sitting time and the presence of anxiety in both men and women are presented in Table 2. In the crude model (Model 1), sitting time was positively associated with the prevalence of anxiety in both men and women (*p* < 0.001), and this association remained significant after sequentially adjusting for the possible confounding factors of age, screening center, alcohol intake, smoking status, education level, history of hypertension, history of diabetes mellitus, LDL-C, weight circumference, vitamin D, and vigorous exercise frequency (*p* < 0.001; Model 2–4). The multivariable-adjusted ORs (95% CI) for the presence of anxiety according to sitting times of 5–9 h/day, and ≥10 h/day, compared to <5 h/day, were 1.034 (0.968–1.106) and 1.136 (1.059–1.220) in men, and 1.093 (1.035–1.155) and 1.231 (1.155–1.311) in women. A longer sitting time was significantly associated with a higher OR for the prevalence of anxiety in women. Based on subgroup analyses by sex, the interaction between sitting time and sex for the prevalence of anxiety was significant in all models, indicating a stronger association in women than in men (*p* < 0.001).

## 4. Discussion

This is the first population study to confirm sex-specific associations between prolonged sitting time and anxiety prevalence. A prolonged sitting time of over 10 h/day was associated with higher anxiety prevalence in both men and women. Particularly noteworthy was the finding that sitting for 5–9 h per day increased the odds of anxiety, especially in women. Overall, women exhibited higher CUXOS scores than men, indicating an increased risk of anxiety with prolonged sitting times.

The average sitting time varies across countries. According to a survey conducted in 62 countries, the median mean daily sitting times was 4.7 h, with higher-income countries recording longer durations than lower-income countries [44]. Notably, major advanced countries have reported even higher values, with the United States having an average sedentary time of 7.7 h per day according to the National Health and Nutrition Examination Survey [45], and four European countries reporting a sedentary time of 8.8 h [46]. In Korea, based on the Korea National Health and Nutrition Examination Survey, the average sedentary time for adults is 7.30 h/day [47], which is similar to the average of 7.4 h observed in this study. Furthermore, men spent more time sitting, evidenced by a sitting time of 7.9 h for men and 6.8 h for women. Additionally, men had higher rates of alcohol consumption and smoking, and a higher prevalence of hypertension, diabetes, hyperlipidemia, and obesity. Previous studies have indicated that an increase in sitting time in men is compounded by various lifestyle risk factors such as smoking, excessive alcohol consumption, and an unhealthy diet, leading to an increased incidence of diseases such as obesity, cardiovascular diseases, type 2 diabetes, and certain cancers [48,49]. In this context, this study highlights the importance of considering and addressing sitting time to improve men’s health.

We demonstrated an association between prolonged sitting time and increased anxiety prevalence in both women and men. Specifically, when sitting time increased to over 10 h, the risk of anxiety increased by approximately 14% for men and 23% for women compared with sitting for less than 5 h. Numerous studies have explored the association between sedentary behavior and mental health, including depression [4,5,50], anxiety [6,10,50], and cognitive impairment [7]. The recent COVID-19 pandemic accelerated the prevalence of sedentary behavior, leading to adverse mental outcomes due to social distancing measures [8,9,29]. However, some studies have found no significant association between these factors in certain populations [51,52]. The relationship between sedentary behavior and anxiety is particularly inconsistent, with significant associations confirmed in large-scale general population studies conducted in the United States, Spain, China, and India [53,54], but some longitudinal studies report no such association [55,56]. In Korea, existing research has been relatively limited, with one study indicating a relatively higher risk of anxiety in the sedentary group (0–600 metabolic equivalents (METs) per min/week) compared with the active group (600–3000 METs per min/week), in the context of the relationship between physical activity and anxiety [57]. Other studies comparing sitting time and anxiety were restricted to specific populations such as adolescents [29], older adults [30], university students [31], and office workers [28], with the added limitation of sitting time being intertwined with screen time [28], and the results being affected by the limitations of the post-COVID-19 period [29]. The strength of this study lies in its confirmation of the association between prolonged sitting time and anxiety in a general population of nearly 150,000 individuals.

An important finding of this study is that the relationship between prolonged sitting time and anxiety prevalence was stronger in women. Even with a slight increase in sitting time, women had a significantly higher risk of anxiety. There are several potential explanations for the sex-specific differences. First, as sitting time increases, screen-based activities may increase, disrupting sleep patterns and activating the central nervous system, leading to the activation of opioids and 5-hydroxytryptamine2c (5-HT2c) receptors, resulting in anxiety [6]. Particularly in women, accumulated sedentary time is associated with increased levels of the pro-inflammatory marker fibrinogen, even after adjusting for physical activity [32]. Additionally, the female sex may act as an amplifier of anxiety induced by screen-based sedentary behavior [26], interpreting physiological changes experienced during physical activity as threatening due to high anxiety sensitivity, potentially leading to the avoidance of such activities [58]. Furthermore, women experienced increased sitting time influenced by factors such as pregnancy, childbirth, child-rearing, and household duties, potentially leading to higher levels of anxiety [21,59]. To validate this correlation, further research is needed that considers these factors among women.

Second, prolonged sitting time threatens physical health, leading to an increase in the prevalence of chronic illnesses, and subsequently, anxiety symptoms. Extended periods of sitting contribute to various metabolic and physiological changes [60], resulting in a higher risk of cardiovascular disease, diabetes, and all-cause mortality [1,2]. The bidirectional association between chronic physical conditions and anxiety disorders is well established [18,19], and conditions such as sarcopenia and osteoporosis further increase the risk of prolonged sitting time [61]. Consistent with our study, previous research has demonstrated that sedentary behaviors have a more detrimental impact on women than on men [1,33].

Finally, according to social withdrawal theory, individuals with high anxiety may avoid anxiety-inducing activities, such as physical activity or face-to-face interaction, leading to social withdrawal and a tendency toward sedentary behavior. This process, accompanied by a decline in self-esteem, may further prompt anxiety symptoms [25]. Previous studies have indicated that anxiety during adolescence increases the risk of sedentary behavior in adulthood [62]. Conversely, a prospective cohort study revealed that sedentary behavior during adolescence influenced an increase in anxiety prevalence six years later [63]. The relationship between sitting time and anxiety is bidirectional, and there may be a vicious cycle in which the outcome negatively affects the cause. The high prevalence of anxiety in women and high anxiety sensitivity may accelerate this cycle [18]. Similar to studies indicating a higher rate of poor mental health in low-income women with high sitting time or social isolation [27], it can be speculated that anxiety associated with social withdrawal may increase in women.

This study has several limitations. First, the cross-sectional study design makes it challenging to establish causality between prolonged sitting time and anxiety prevalence. Second, participants in a relatively healthy health checkup program may not represent the entire South Korean population. Therefore, anxiety prevalence in this study might be lower than that established in previous studies and may not accurately reflect actual rates. Third, the data for this study were collected from a single institution between 2012 and 2019, which introduces selection bias and necessitates careful interpretation of the findings. However, this fact led to exploring the direct relationship between sitting time and anxiety, excluding the biological and psychosocial effects triggered by the COVID-19 pandemic that emerged after 2020. Fourth, this study did not differentiate between prolonged sitting time based on context, such as work, commuting, or hobby-related activities. Further research is needed to investigate whether there are differences in anxiety prevalence based on these variables. Finally, factors such as low estrogen levels or menopausal status in women could be associated with bone and muscle loss and may significantly impact anxiety prevalence. Therefore, additional research that considers other contributing factors, such as leptin, estrogen, and menopausal status, is necessary.

## 5. Conclusions

This study revealed a significant positive association between prolonged sitting time and anxiety prevalence, which was more pronounced in women than in men. In particular, the findings highlight the increased vulnerability of women to prolonged sitting times, suggesting the need for sex-specific differentiation in recommendations for optimal sitting time, targeting groups that are at high risk for anxiety. This underscores the importance of public health interventions and emphasizes the necessity to support women in engaging in more physical activity. Future research should utilize population-based, longitudinal studies to explore whether prolonged sitting time is a strong risk factor for anxiety disorders and determine optimal sitting times across the lifespan differentiated by sex.

## Figures and Tables

**Table 1:** Baseline characteristics of the study population by sex.

Characteristics	Total	Men	Women	* *p* Value
Number of subjects (n)	149,772	85,636	64,136	
Screening center, Seoul (%)	47.5	46.3	49.2	<0.001
Age (years)	41.2 ± 9.3	41.6 ± 9.2	40.5 ± 9.4	<0.001
Height (cm)	168.2 ± 8.5	173.7 ± 5.8	160.9 ± 5.3	<0.001
Weight (kg)	67.4 ± 13.5	75.3 ± 10.8	56.8 ± 8.7	<0.001
Waist circumference (cm)	81.7 ± 10.0	86.5 ± 8.1	75.1 ± 8.4	<0.001
Fat mass (kg)	17.9 ± 6.2	18.2 ± 6.3	17.4 ± 6.0	<0.001
Percent body fat (%)	26.4 ± 6.6	23.7 ± 5.5	30.1 ± 6.2	<0.001
ASM (kg)	20.8 ± 4.9	24.5 ± 3.0	16.0 ± 2.1	<0.001
Current smoker (%)	13.7	22.7	1.6	<0.001
Heavy drinking ^a^ (%)	9.9	13.9	5.7	<0.001
High education level ^b^ (%)	62.7	75.6	84.9	<0.001
Sitting time (h/day)	7.4 ± 3.5	7.9 ± 3.4	6.8 ± 3.6	<0.001
Vigorous exercise (days/week)	0.93 ± 1.5	1.1 ± 1.5	0.7 ± 1.4	<0.001
Comorbidities				
Hypertension (%)	12.6	17.7	5.8	<0.001
Diabetes mellitus (%)	3	4.1	1.5	<0.001
Dyslipidemia (%)	16.5	22.1	9.1	<0.001
Laboratory findings				
Vit D (ng/mL)	16.9 ± 7.1	17.1 ± 6.7	16.5 ± 7.6	<0.001
Total cholesterol (mg/dL)	189.4 ± 33.8	192.7 ± 34.3	185.0 ± 32.5	<0.001
LDL-C (mg/dL)	128.3 ± 33.2	134.5 ± 33.0	120.0 ± 31.5	<0.001
HDL-C (mg/dL)	60.7 ± 16.3	54.5 ± 13.5	69.0 ± 16.0	<0.001
Triglycerides (mg/dL)	116.0 ± 81.2	137.3 ± 91.8	87.6 ± 52.7	<0.001
Fasting glucose (mg/dL)	97.2 ± 14.7	100.0 ± 15.8	93.6 ± 12.1	<0.001
HbA1c (%)	5.5 ± 0.5	5.6 ± 0.6	5.4 ± 0.4	<0.001
Creatinine (mg/dL)	0.84 ± 0.22	0.96 ± 0.20	0.69 ± 0.13	<0.001
Albumin (g/dL)	4.81 ± 0.25	4.88 ± 0.23	4.72 ± 0.25	<0.001
AST (IU/L)	22.9 ± 13.9	25.5 ± 15.6	19.5 ± 10.3	<0.001

Values are mean ± SD or percentage. * Using chi-square test for categorical variable or Student’s *t*-test for continuous variable. ^a^ Men ≥ 30 g/day, women ≥ 20 g/day. ^b^ ≥college graduate. ASM, appendicular skeletal muscle mass; vit D, vitamin D; LDL-C, low-density lipoprotein cholesterol; HDL-C, high-density lipoprotein cholesterol; HbA1c, hemoglobin A1c; AST, aspartate aminotransferase.

**Table 2:** Association of sitting time with presence of anxiety by sex.

	Sitting Time				
	<5 h/day, OR (95% CI)	5–9 h/day, OR (95% CI)	≥10 h/day, OR (95% CI)	*p* for Trend	*p* for Interaction (Men vs. Women)
Men					
Model 1	1 (reference)	0.976 (0.929–1.025)	1.079 (1.025–1.136)	<0.001	<0.001
Model 2	1 (reference)	1.062 (1.003–1.125)	1.203 (1.132–1.279)	<0.001	<0.001
Model 3	1 (reference)	1.045 (0.978–1.117)	1.159 (1.081–1.244)	<0.001	<0.001
Model 4	1 (reference)	1.034 (0.968–1.106)	1.136 (1.059–1.220)	<0.001	<0.001
Women					
Model 1	1 (reference)	1.143 (1.098–1.189)	1.370 (1.310–1.432)	<0.001	
Model 2	1 (reference)	1.120 (1.068–1.175)	1.326 (1.256–1.401)	<0.001	
Model 3	1 (reference)	1.109 (1.050–1.172)	1.257 (1.181–1.339)	<0.001	
Model 4	1 (reference)	1.093 (1.035–1.155)	1.231 (1.155–1.311)	<0.001	

Model 1: crude; Model 2: adjusted for age, screening center, alcohol intake, smoking status, and education level; Model 3: adjusted for age, screening center, alcohol intake, smoking status, education level, history of hypertension, history of diabetes mellitus, LDL-C, waist circumference, and vit D; Model 4: adjusted for age, screening center, alcohol intake, smoking status, education level, history of hypertension, history of diabetes mellitus, LDL-C, waist circumference, vit D, and vigorous exercise frequency. Abbreviations: vit D, vitamin D; LDL-C, low-density lipoprotein cholesterol; CI, confidence interval; and OR, odds ratio.

## Data Availability

The data that support the findings of this study are available from the corresponding authors upon reasonable request. The data are not publicly available due to ethical restrictions that protect patient privacy and consent.
